# Supplementary figures and images for: Control of Paneth cell function by HuR regulates gut mucosal growth by altering stem cell activity
Source: Life Sci Alliance. 2023 Sep 11;6(11):e202302152. doi: 10.26508/lsa.202302152 (PMC10494932; doi:10.26508/lsa.202302152)

**Figure 4B: Cyto-C**

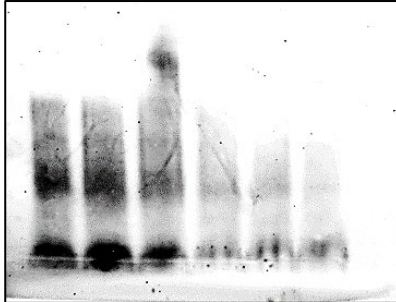

**Figure 6F: PHB2**

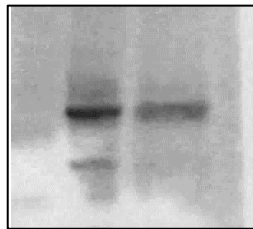

Supplement: Supplementary file 1 [file LSA-2023-02152_SdataF4_F6.pdf]
